# Short term cost effectiveness of a regional myocardial infarction network

**DOI:** 10.1186/2191-1991-3-10

**Published:** 2013-04-08

**Authors:** Ralf Birkemeyer, Anke Dauch, Alfred Müller, Manfred Beck, Henrik Schneider, Hueseyin Ince, Werner Jung, Steffen Wahler

**Affiliations:** 1 Heart Center Rostock, Universitätsklinikum Rostock, Ernst-Heydemann-Str. 6, 18057, Rostock, Germany; 2 Department of Cardiology, Schwarzwald-Baar-Klinikum, Villingen-Schwenningen, Germany; 3 Analytic Services, Munich, Germany; 4 Universitätsklinikum Tübingen, Tübingen, Germany; 5 Fachhochschule für Gesundheit und Medizin, Hamburg, Germany

**Keywords:** STEMI, Network, Primary PCI, Cost analysis, Cost effectiveness

## Abstract

**Aims:**

Myocardial infarction networks have been shown to improve guideline adherent therapy and outcomes in patients presenting with acute ST-elevation myocardial infarction (STEMI). Our objective was to assess the short term cost effectiveness of a network structure.

**Methods and results:**

Outcome data and reimbursement data for the index hospital stay were gathered in consecutive patients with acute STEMI (n = 536) admitted to any of the hospitals in a 350.000 inhabitant rural network area during the years 2002 (n = 185), 2005 (n = 163) and 2008 (n = 188). Network structure was established between 2002 and 2005 aiming for identical treatment of all acute STEMI patients during 24 h/7d a week with primary angioplasty. Patient baseline characteristics in the different years were quite comparable. From 2002 to 2005 regional hospital mortality in STEMI patients decreased from 16% to 9%. Lower mortality under network conditions was confirmed in 2008. Reimbursement data of different years were standardized to exclude effects not induced by the network. The mean initial costs per saved live during the index stay were €7727 with a 95%-confidence interval of €-3.500 to €36.700 (referenced to the German reimbursement in 2005).

**Conclusion:**

The short term cost effectiveness of a myocardial infarction network organisation is within well accepted boundaries under conditions of the German reimbursement system.

## Background

After a steep increase in infarction mortality in the nineteen-fifties and nineteen-sixties myocardial infarction became the most frequent cause of death world wide. Since the nineteen-nineties infarction mortality is on the decline again. This was induced by behavioural changes with respect to modifiable risk factors and a better acute and chronic therapy for patients presenting with acute myocardial infarction. A cornerstone of the acute infarction therapy is timely reperfusion of the infarct-related artery either performed pharmacologically or mechanically. Timely reperfusion is especially demanded for ST-elevation myocardial infarction (STEMI) which is an entity of myocardial infarctions that can be readily identified by well-defined criteria in the rest electrocardiogram (ECG) and clinical symptoms. There are only a few conditions which mimic the ECG changes and symptoms of a STEMI. These so called masquerading STEMI’s usually represent less than 5% of the initially diagnosed STEMI patients [1].

Primary percutaneous coronary intervention (PCI) is the mechanical reperfusion method in myocardial infarction. It is superior to pharmacological reperfusion with fibrinolysis in patients presenting with acute ST-elevation myocardial infarction (STEMI) when it can be performed expeditiously by an experienced team in a hospital with an established interventional cardiology programme (24 h/7 days) and the PCI related time delay compared to fibrinolysis is no longer than 90–120 minutes [2-8]. Lowest mortality rates among patients undergoing primary PCI are observed in centres with a high volume of PCI procedures [9,10]. Therefore optimal treatment of STEMI should be based on the implementation of a network between hospitals with various levels of technology, connected by an efficient ambulance or helicopter service [2,11].

Very encouraging results from different registries have recently been published which show that implementation of myocardial infarction network structures in fact promotes guideline adherent therapy and reduces mortality [12-18]. Patients at high risk seem to profit most [12,18].

Whereas some data exist on the cost effectiveness of primary PCI compared to fibrinolysis [19-21] such data lack for the implementation of an emergency medical services-based routine transfer for primary PCI compared to standard care with concomitant use of transfer PCI and fibrinolysis at the discretion of the local hospital. Emergency medical services-based routine transfer was identified as the most cost-effective approach to promote access to primary PCI in a simulation of different strategies in the U.S. American setting [22].

Routine transfer PCI however might have an underestimated impact on short term health care expenditures due to better access of critically ill STEMI patients to complex intensive care treatment including mechanical circulatory support.

Therefore we collected and analyzed reimbursement data in a regional all comer registry in the last year before and the first year after network implementation. Consistency of the network induced changes was checked by an additional data collection three years after.

## Methods

### Organization of the regional network

The network serves a rural population of approximately 350,000 inhabitants. The diameter of the network region is up to 70 km. There are six public regional hospitals, five of them without cathlabs and one with a high-volume interventional facility and a 24 h/7d primary PCI service.

Network structures include 12-lead ECG in the ambulance, ECG telemetry to the intensive care unit of the invasive facility, a structured phone call between emergency medical services (EMS) and the intensive care physician on call and preparation of the cathlab before patient arrival. STEMI patients are intended to be directly admitted to the cathlab, irrespective of the presence of cardiogenic shock or resuscitation.

The uniform, regional primary PCI protocol aims for identical treatment of all acute STEMI patients during 24 h/7d a week in one interventional centre. In a population based analysis it was already shown that approximately 90% of patients with acute STEMI being admitted to any of the hospitals in the network area received primary PCI [18]. Nearly all patients who were not scheduled for primary PCI were excluded from any revascularisation attempt due to co-morbidities.

### Study population and follow up

All consecutive patients with the clinical diagnosis of acute ST-elevation myocardial infarction (symptom duration <12 hours) admitted to any of the network area hospitals during the years 2002 (n = 185), 2005 (n = 163) and 2008 (n = 188) were included in this registry irrespective of their treatment. The network was established in between 2002 and 2005.

All patients in the invasive facility were prospectively documented in a dedicated database. Completeness was checked by comparison with the compulsory ICD-10 encoding of the clinical diagnoses in the hospital discharge dataset (code I21.X with exclusion of non ST-elevation myocardial infarctions). All patients treated exclusively in the non cathlab hospitals were retrospectively identified by their ICD-10 encoding as described above. Clinical files of those patients were subsequently checked by one clinician. Reimbursement data on the index stay for each single patient was obtained from hospital databases. Reimbursement for cardiac surgery was included in the analysis if patients were directly transferred for cardiac surgery.

Follow up was obtained by telephone interviews and questionnaires at 6 months with complete information on mortality gathered from state registries. The registry was approved by the local ethics committee. All patients were asked for their informed consent for the extension of the institutional routine follow-up.

### Assessment of quality of life after primary PCI under network conditions

From January 2005 to mid 2007 about 500 hundred STEMI patients were scheduled for primary PCI under network conditions. These consecutive patients received the German version of the MacNew heart disease health-related quality of life questionnaire at the time points 30 days, 6 months and 12 months after the angiography. 298 patients returned the questionnaire.

The MacNew is a disease specific instrument for patients with cardiac disease describing with 3 scales the physical, emotional and social quality of life. In addition a global quality of life value can be calculated. Each scale ranges from 1 to 7 with 1 indicating a poor and 7 a perfect quality of life. The instrument is not yet validated for the calculation of QALY’s.

The purpose of our repeated measurements with this instrument was to determine how fast patients return to a stabilized health status after primary PCI.

### Reimbursement comparison

In order to identify the impact of the network structure implementation on reimbursement subsequent years had to be compared. Accordingly the impact of other structural and price changes between years had to be eliminated. In this respect the change of the reimbursement system from the German Fallpauschalensystem (lump compensation system) to the G-DRG (German Diagnosis Related Groups) system after 2002 was a major challenge.

### Standardization of reimbursement by a relative weight model

There was no established methodology for comparison of health expenditures before and after transition from the German Fallpauschalen to the G-DRG system. Use of the 2005 G-DRG grouper for the entire patient cohort seemed to be an appealing concept to stratify patients according to severity of disease manifestation, extent of required medical procedures and co morbidities following identical rules. An increase of the mean relative weight from 2002 to 2005 respectively 2008 in patient cohorts with comparable baseline characteristics should be an appropriate indicator for the change in medical care driven by the network organization. Furthermore this could be easily translated into comparable mean reimbursement for patient treatment in different years. It was however soon evident that the available data documentation of the 2002 patients was not suitable for standard grouping.

Therefore a simplified stratification system was created based on the clinical and procedural information available for each patient leading into one of six reimbursement groups comparable to the main G-DRG’s for STEMI patients in 2005. The algorithm used checked for the duration of intensive care stay (decision criteria: > 5 days), presence of cardiogenic shock, initial resuscitation, use of an intra aortic counterpulsation (IABP), performance of PCI, amount of stents implanted, interhospital transfer and transfer for coronary artery bypass grafting (CABG). If patients got more than one stent implanted they were stratified into the “complex PCI” group, if patients stayed more than five days on intensive care and/or had a cardiogenic shock and/or an initial resuscitation an/or an IABP implanted they were considered as receiving “complex care”. Assumed relative weights for each reimbursement group were derived from the 2005 STEMI G-DRG’s. For patients with inter hospital transfer (hub and spoke) two separate relative weights for the interventional and the peripheral stay were determined (with a 40% discount on the interventional and a 30% discount on the peripheral relative weight to compensate for reimbursement cuts due to short stays). For performance of CABG an assumed relative weight of 5.5 was added (also derived from the 2005 G-DRG catalogue). Use of drug eluting stents was not included in the relative weight model.

All 536 registry patients of different years were stratified in one of the reimbursement groups according to the described algorithm. Further stratification was done according to inter hospital transfer (hub and spoke) and transfer for CABG. One patient in the “Angiography only” group had an excessively high reimbursement compared to the mean of the group and was excluded from further analysis. The mean relative weight for a STEMI patient treated in the years 2002, 2005 and 2008 was determined. Afterwards the mean relative weight was multiplied with the real base rate (€2855) valid for the network area in 2005 leading to a standardized mean reimbursement received for a STEMI patient in the network area in the respective years. After further standardization to a hypothetical patient number of 180 patients overall reimbursements per year could be compared. Cost deltas were calculated.

### Transportation and other costs

Transportation costs were not included in the final analysis, as they are highly variable within Germany and reimbursement rules vary a lot between countries. Actually numbers of overall and reimbursed EMS transfers decreased with network implementation.

Before the network about 70% of patients from the catchment area of the non-invasive hospitals had an EMS-escorted transfer to the peripheral hospital and one third an early EMS-escorted transfer from the peripheral hospital to primary PCI. In addition some peripheral patients were transferred late during their hospital stay for coronary angiography and also some transferred back. With the network implementation the number of initial EMS transfers to the peripheral hospital markedly decreased whereas the number of direct transfers to the invasive facility from the catchment area of the non-invasive hospitals increased as did the number of back transfers to the peripheral hospitals.

In the German reimbursement system each local EMS has a standard fee for an ambulance transfer independent of distance which only differs according to the fact that an emergency physician is aboard or not; costs for airbound transport can differ but this option was rarely used; back transfers to lower level hospitals are in general not reimbursed at all and are therefore organized and paid by the invasive facility. The uncorrected number of reimbursed EMS escorted transfers dropped from 2002 to 2005 by 47 respectively from 2002 to 2008 by 31. The average costs for an EMS-escorted transfer at that time were about €400 in the network area leading to an uncorrected overall cost decrease of about €19.000 respectively €12.000 in 2005 and 2008. If back transfers would have been reimbursed the major part of the cost saving would have disappeared, even more after standardization for patients numbers.

Networks have no direct reimbursement effects in the German system. Costs for necessary organisational changes and running of the network were anyway negligible. All meetings were organized by the representatives of the different organisations during their working hours within the hospital facilities. The biggest part of communication was done electronically. No dedicated staff was hired for the project. The existing technical equipment fulfilled the needs.

Expansion of the reimbursement analysis to the post discharge period was not possible because of lack of access to ambulatory data. Decision was made to renounce on further modelling.

### Source of change analysis

For crosscheck of the relative weight model a top down source of change analysis was performed including the following steps: summation of real per patient reimbursement stratified according to year and reimbursement group; inclusion of CABG; standardization to 180 patients per year, standardization of reimbursement 2002 and 2008 to the 2005 level, calculation of base rates (division of the standardized overall reimbursement per year by the mean relative weight) with correction for drug eluting stent use (extra reimbursement).

### Cost effectiveness analysis

After calculation of the reimbursement changes attributable to the network implementation a cost effectiveness analysis was performed. The primary endpoint considered was mean costs per saved life with the 95%-confidence intervals computed using nonparametric bootstrapping.

### Statistical methods

Data were analyzed according to established standards of descriptive statistics. Categorical variables were compared by a maximum likelihood χ^2^ test. Continuous variables are reported as medians with interquartile range or mean ± SD. For comparisons, the *t* test (based on testing for normal distribution) or the 2-tailed Mann–Whitney *U* test was used as appropriate. Odds ratios and 95% confidence intervals were provided where appropriate. A p-value of less than 0.05 was considered significant. Bootstrapping was done with 5000 case resamplings.

## Results

Five hundred and thirty six consecutive patients (n = 536) with the diagnosis of acute STEMI (<12 h) were included in this area-wide registry irrespective of their treatment: 185 patients in 2002 (last year before the myocardial infarction network was established), 163 patients in 2005 (first year of complete network implementation) and 186 patients in 2008 (“chronic” network structures).

Patients’ baseline characteristics were quite comparable between different years (Table 1). There was however a significantly higher proportion of females in 2002 and a trend over time to more patients being admitted in shock and after resuscitation.

**Table 1:** Baseline clinical characteristics of acute regional STEMI patients according to the year of hospital admission

	**2002 (n = 185)**	**2005 (n = 163)**	**2008 (n = 188)**	**p value**
Age (yrs.)	67 ± 13	66 ± 13	63 ± 14	n.s.
Female gender	41%	29%	27%	0.01
Diabetes	29%	24%	26%	n.s.
Current smoker	33%	39%	35%	n.s.
Arterial hypertension	69%	61%	68%	n.s.
Hyperlipidemia	63%	45%	46%	<0.01
Previous myocardial infarction	12%	9%	10%	n.s.
Previous PCI	4%	7%	6%	n.s.
Previous CABG	1%	2%	2%	n.s.
Cardiogenic shock	11%	13%	15%	n.s.
Post CPR	6%	9%	12%	n.s.

Data are presented as mean value ± SD or percentage of patients.

*CABG*, Coronary bypass graft; *CPR*, Cardiopulmonary resuscitation; *n.s*, Non significant; *PCI*, Percutaneous coronary intervention.

Implementation of the network structures led to different utilization of reperfusion and reperfusion methods. In 2002 27% of patients received fibrinolysis and 53% primary PCI while 21% of patients received no early reperfusion treatment at all (including the 3% of patients with aborted PCI). In 2005 this had changed to 2% fibrinolysis, 89% primary PCI and 9% without reperfusion treatment. These results have been previously published [18]. In our present analysis the network effect was confirmed in 2008 with 1% fibrinolysis, 89% primary PCI and 10% without reperfusion treatment.

Regional mortality for STEMI differed considerably between 2002 and 2005: at hospital discharge the difference was 7% (16% versus 9%), which increased to 9% (19% versus 10%) after 6 month. This was mainly driven by a mortality reduction in the elderly and shock patients [17]. The mortality effect was confirmed in 2008 with a hospital mortality of 7% and a 6 month mortality of 10% (Table 2).

**Table 2:** Treatment strategies and outcome in acute regional STEMI patients according to the year of hospital admission

	**2002 (n = 185)**	**2005 (n = 163)**	**2008 (n = 188)**	**p value**
No immediate revascularisation	21%	9%	10%	<0.01
Fibrinolysis	27%	2%	1%	0.01
Primary PCI	53%	89%	89%	<0.01
Hospital mortality	16%	9%	7%	0.01
6-month mortality	19%	10%	10%	0,01

No immediate revascularisation also includes intended but aborted primary PCI’s.

Repeated measurements with the MacNew quality of life questionnaire showed that STEMI patients regained a stable health status within 30 days after primary PCI which then basically remained unchanged up to 12 months (Table 3). Only the elderly (> 65 years) had a trend for deterioration between 30 days and 6 months from which they recovered up to 12 months.

**Table 3:** Subjective disease related quality of life (QoL) in 298 patients at different time points after primary PCI (according to the MacNew questionnaire)

	**30 days mean±SD**	**6 months mean±SD**	**12 months mean±SD**	**p-value t1-t2**	**p-value t2-t3**	**p-value t1-t3**
Global QoL	5.1 ± 1.1	5.2±1.2	5.3±1.1	.318	.312	.072
Physical QoL	5.1±1.2	5.2±1.3	5.3±1.2	.473	.170	.111
Emotional QoL	5.1±1.2	5.1±1.2	5.2±1.1	.391	.854	.248
Social QoL	5.2±1.2	5.4±1.3	5.4±1.2	.063	.357	.014

Scales range from 1 to 7 with 1 representing poor and 7 perfect health.

Table 4 displays the results of the patient stratification into the relative weight model. Most striking is the jump in the number of patients requiring complex care from 2002 to 2005 in the PCI centre. This represents a change in the pattern of care for shock and post resuscitation patients from a medical stabilization attempt in the nearest hospital to an early transfer for immediate mechanical revascularisation. In addition more patients received complex PCI with less patients being transferred for CABG. An increase of approximately 10% of the mean relative weight respectively mean reimbursement for a network area STEMI patient from 2002 to 2008 seems to be attributable to network effects.

**Table 4:** Patient stratification into the relative weight model

						**Relative weight hub**	**Relative weight spoke**
	**Reimbursement stratum**	**CABG**	**Year**				
**Hub only**			**2002**	**2005**	**2008**	**1**	**0**
	A: Complex care		9	24	30	3.308	
	B: Complex PCI		25	34	54	2.115	
	C: Non-complex PCI		37	49	38	1.874	
	D: Angiography. only		8	4	8	2.917	
	E: No angiography		1	3	5	1.421	
			*80*	*114*	*135*	**0.6**	**0.7**
**Transfer**	A: Complex care	CABG	3	1	2	1.985	3.85
		Cons.	5	2	2	1.985	0.805
	B: Complex PCI	CABG	4	3	3	1.269	3.85
		Cons.	5	21	22	1.269	0.805
	C: Non-complex PCI	CABG	1			1,246	3.85
		Cons.	27	15	15	1.246	0.805
	D: Angiography only	CABG	3			1.750	3.85
		Cons.	8		2	1.750	
	E: No angiography	Cons.	4	1	1	0.852	
			*60*	*43*	*47*	**0**	**1**
**Spoke only**	X: External	CABG	1				5.50
		Cons.	33	6	6		1.15
		PCI (not in hub)	11				1.93
			*45*	*6*	*6*		
			***185***	***163***	***188***		
**Mean relative weight**		**2.135**	**2.246**	**2.352**			
Change		0.951	1	1.047			

The comparison of the mean reimbursement for a STEMI patient in 2005 derived from the model (mean relative weight multiplied with the real base rate 2005) with the real reimbursement served as a quality indicator of the relative weight model. The mean modelled reimbursement (without DES) in 2005 was €6412, the mean real reimbursement was €6055 (plus €578 for DES).

As first step of the source of change analysis real per patient reimbursement data plus estimated CABG costs per year were summed up. Then the real cost sums of 2002 and 2008 were standardized to the 2005 reimbursement level. Third step was standardisation to a hypothetical patient number of 180 patients per year. Afterwards mean reimbursement was calculated for each different year. After correction for different DES use (extra reimbursement) the cost delta between 2002 and 2008 was matching the result of the relative weight model. As a quality indicator for the top down source of change analysis a virtual base rate was calculated (mean standardized reimbursement divided by mean modelled relative weight 2005) and compared to the real base rate 2005. The virtual base rate (after correction for DES use) was €2534, the real base rate €2855.

**Figure 1:**
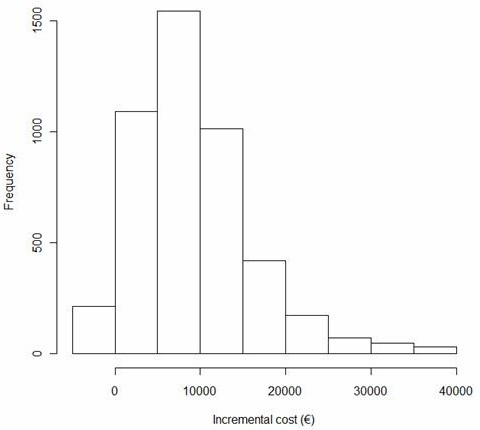
Distribution of incremental costs per saved life during index stay (reference: 2005 reimbursement) for an emergency medical services-based strategy of transporting every patient to primary PCI; result of a bootstrap analysis with 5000 re-samplings.

The 10% increase in reimbursement for the index hospital stay and the observed mean 8% decrease in mortality at discharge after network implementation translated into short term mean costs per saved life of €7727 (reference: 2005 German reimbursement level). A 95%-confidence interval of €-3.500 to 36.700 was computed using non parametric bootstrapping (Figure 1).

## Discussion

Implementation of a myocardial infarction network in a rural German surrounding aiming at identical treatment of all consecutive STEMI patients with transfer to primary PCI increased mean health care expenditures for the index hospital stay by approximately 10%. Increased expenditures were mainly driven by the higher number of patients receiving complex intensive care after STEMI complicated by cardiac arrest and/or shock. In addition a higher number of complex PCI procedures was performed which was paralleled by a decrease in the number of patients with early post myocardial infarction CABG. Transportation costs were not considered in this analysis as they are highly variable even within the German reimbursement system. If considered they would have attenuated the network effect on local health care expenditures by 1-2%.

The population based registry showed an absolute decrease of STEMI mortality after network implementation of mean 8% at hospital discharge and 9% at 6 month. The increase of the mortality benefit over time fits with the long term follow up of a randomized trial comparing primary PCI with fibrinolysis where the mortality difference of 6% after 30 days increased to 11% after a mean follow up of 5 years [23].

Before network implementation reperfusion therapy was performed at the discretion of the nearest emergency department with concomitant use of transfer PCI and fibrinolysis. The observed regional mortality of 16% at that time matches well the data from the population based WHO MONICA (monitoring trends and determinants in cardiovascular disease) and its successor project KORA (Kooperative Gesundheitsforschung in der Region Augsburg) [24,25], which found an overall hospital infarction mortality of approximately 17% in the years 2001 to 2003. On the other hand the decrease of hospital mortality to mean 8% after implementation of the network fits well with observations from other network registries in Austria, Czech and Italy [12,16,17].

Our findings translate into incremental costs of €7727 per saved life during index hospital stay (95% CI €-3.500 to €36.700) attributable to the myocardial infarction network (referenced to 2005 German reimbursement).

For comparability reasons the magnitude of gained quality adjusted life years (QALY) due to the network implementation can be estimated. The 6 month mortality reduction already leads to an approximate gain of 8 unadjusted life years in a cohort of 180 patients during this short time span. It is known that the self reported quality of life in patients having stabilized after acute coronary syndromes is comparable to patients with stable coronary artery disease that never experienced a myocardial infarction [26,27]. The health utility score (EQ-5D index, US) in a large cohort of coronary heart disease patients with stable health status was reported to be approximately 0.7 [27] which seems to correspond well to the quality of life reflected by the indices we have measured with the MacNew instrument already in the early post interventional time course [26]. Taking this into consideration already the effect of early mortality reduction could account for as much as 5.6 QALY’s. Furthermore the general replacement of fibrinolysis by primary PCI leads to a lower number of reinfarctions and strokes translating into an additional positive effect on QALY’s gained [22]. Even though we have no data on health care expenditures after hospital discharge in our study cohort it seems reasonable to assume that the cost delta does not continue to increase with more expected therapy associated complications after fibrinolysis compared to documented early stabilization of health status in the primary PCI group. Considering only the early gain this would translate into costs of about 20.000 €/QALY.

Obviously this number is very speculative and comparability of this finding with the results of an American cost simulation estimating the costs per QALY for establishing an emergency medical services-based strategy of routine transfer PCI therefore limited. The observed magnitude in our registry seems to be higher than the estimated $506 (95% CI $474 to $519) in the simulation [22]. However under the assumption of a persisting mortality benefit in an acceptable health status over five years the simulated results seem to be reasonable.

Furthermore it seems reasonable to assume that the incremental cost effectiveness ratio of our network organisation is well below the often cited willingness-to-pay threshold of €80.000/QALY; and also below the estimated incremental cost effectiveness ratio of approximately €44.000 Euro/QALY of primary prophylactic ICD therapy in patients with a left ventricular ejection fraction < 40% [28].

### Limitations

This study suffers the inherent problems of any observational study. However, we included all consecutive patients admitted with the same diagnosis in the same territorial area and hospitals in three subsequent time frames. So we got a comprehensive view on the effect of the modification of our regional treatment concepts on the outcome of our infarction population.

Lack of ambulatory cost data definitely is a major limitation; in addition primary PCI patients were followed with a disease specific quality of life questionnaire (German version of the MacNew) which is not validated for calculation of QALY’s.

Further limitations are the retrospective identification of the subset of patients treated conservatively in the non-invasive hospitals, the sample size which limits the power of any analysis and the assumptions that had to be made for cost comparison between different years and reimbursement systems.

## Conclusions

Our data confirm that the short term cost effectiveness of a German myocardial infarction network organisation aiming for primary PCI in all STEMI patients is within well accepted boundaries.

## Competing interests

The authors declared that they have no competing interest.

## Authors’ contributions

RB and AD participated in the design of the study as well as the data collection and drafted the manuscript. AM participated in the design of the study, did the statistical analysis and revised the manuscript. MB, HS, HI and WJ carefully revised the design of the study and the final manuscript. All authors read and approved the final manuscript.
